# Nanobiosensing with Arrays and Ensembles of Nanoelectrodes

**DOI:** 10.3390/s17010065

**Published:** 2016-12-30

**Authors:** Najmeh Karimian, Ligia M. Moretto, Paolo Ugo

**Affiliations:** Department of Molecular Sciences and Nanosystems, University Ca’ Foscari of Venice, Via Torino 155–Mestre, 30172 Venice, Italy; najmeh.karimian@unive.it (N.K.); moretto@unive.it (L.M.M.)

**Keywords:** nanoelectrode, ensemble, array, voltammetry, biosensor, bioelectroanalysis

## Abstract

Since the first reports dating back to the mid-1990s, ensembles and arrays of nanoelectrodes (NEEs and NEAs, respectively) have gained an important role as advanced electroanalytical tools thank to their unique characteristics which include, among others, dramatically improved signal/noise ratios, enhanced mass transport and suitability for extreme miniaturization. From the year 2000 onward, these properties have been exploited to develop electrochemical biosensors in which the surfaces of NEEs/NEAs have been functionalized with biorecognition layers using immobilization modes able to take the maximum advantage from the special morphology and composite nature of their surface. This paper presents an updated overview of this field. It consists of two parts. In the first, we discuss nanofabrication methods and the principles of functioning of NEEs/NEAs, focusing, in particular, on those features which are important for the development of highly sensitive and miniaturized biosensors. In the second part, we review literature references dealing the bioanalytical and biosensing applications of sensors based on biofunctionalized arrays/ensembles of nanoelectrodes, focusing our attention on the most recent advances, published in the last five years. The goal of this review is both to furnish fundamental knowledge to researchers starting their activity in this field and provide critical information on recent achievements which can stimulate new ideas for future developments to experienced scientists.

## 1. Introduction

Owing to their characteristics of high sensitivity, compactness and easy integration with other analytical devices, arrays of nanoelectrodes offer great potential for bioanalytical applications [1,2,3]. In particular, the development of biosensing devices based on arrays of nanoelectrodes [4] in the form of nanodisks, nanowires, nanochannels and nanopores presents unique perspectives for the screening and detection at ultrahigh sensitivities of analytes of biological interest, which can include both biomacromolecules (e.g., proteins, polynucleotides) and small molecules (e.g., drugs, metabolites, toxic ions). Here we will use the definition nanoelectrode array (NEAs) and ensemble (NEE) to distinguish ordered from random arrangements of nanoelectrodes, respectively. From a general viewpoint, low cost, miniaturizability, easy use, no interference from colored or turbid matrices, applicability to raw samples for “in situ” and decentralized monitoring distinguish bioelectrochemical sensors with respect to classical instrumental methods such as spectroscopy, chromatography and mass-spectrometry. The nanostructuration of the sensor surface contributes in increasing the specific area available for the immobilization of high amounts of the biomolecules involved in the recognition event, while keeping the overall size of the sensor to very small figures [5,6,7]. Moreover, it is possible to engineer the sensor surface to separate, at the nanoscale range, the biorecognition and transduction events [8,9]. 

After a general introduction on the preparation, characterization and properties of NEEs/NEAs, this review focuses on current progresses on the analytical and biological application of NEEs/NEAs-based sensors. Attention is put in particular on arrays of metal or semiconductor nanoelectrodes. The quite broad topic concerning arrays of nanoelectrodes produced using carbon nanotubes alignment and similar procedures is beyond the scope of the present review. Readers interested in this topic are referred to specific articles and reviews, see e.g., [10,11,12,13,14,15,16,17,18,19].

## 2. Template Ensemble of Nanoelectrodes

**Figure 1:**
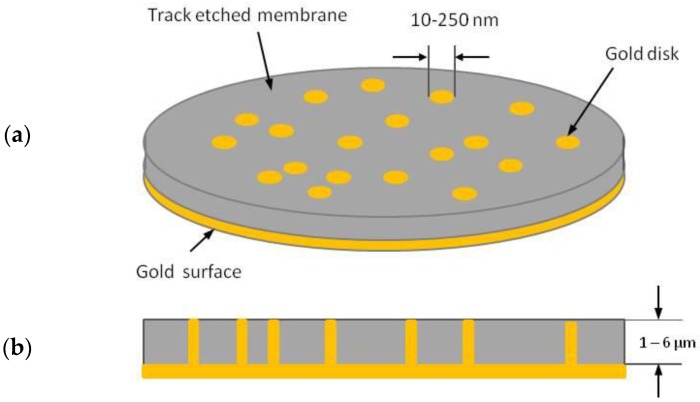
Schematic diagram of a nanoelectrode ensemble in a template membrane: (**a**) overall view; (**b**) cross section (redrawn and reprinted with permission from [4]).

The development of templated NEEs has made accessible to almost any chemical laboratory the preparation and use of nanoelectrodes. The first template synthesis of NEEs for electrochemical use was described by Menon and Martin [20], who deposited gold nanofibres with a diameter as small as 10 nm within the pores of track etched polycarbonate (PC) membranes by a chemical (electroless) method, obtaining a random ensemble of metal nanodisk electrodes surrounded by the insulating polymer. All the nanoelectrodes were interconnected to each other so that they all experienced the same electrochemical potential (see Figure 1). 

In the membrane template synthesis the pores of a host material are used to direct the growth of new materials, typically gold and, later, other metals [20,21,22]. Various examples of membrane templated electrochemical deposition of nanowires of semiconductors [23], metals (e.g., Ni and Co) [24], oxides and conducting polymers [1] have appeared in the literature. The metal fibers growth can be performed both by using electrochemical [24,25] or electroless [20,26,27] deposition methods.

In template deposition, the pore density in the template determines the number of metal nanoelectrode elements on the NEE surface and, correspondingly, the average distance between them, while the diameter of the pores in the template determines the diameter of the individual nanoelectrodes. Track-etched membranes with different pore diameters (e.g., 10, 30, 200 nm) and average pore distance of ≥200 nm are commercially available.

### 2.1. Template Electrochemical Deposition of Metals

**Figure 2:**
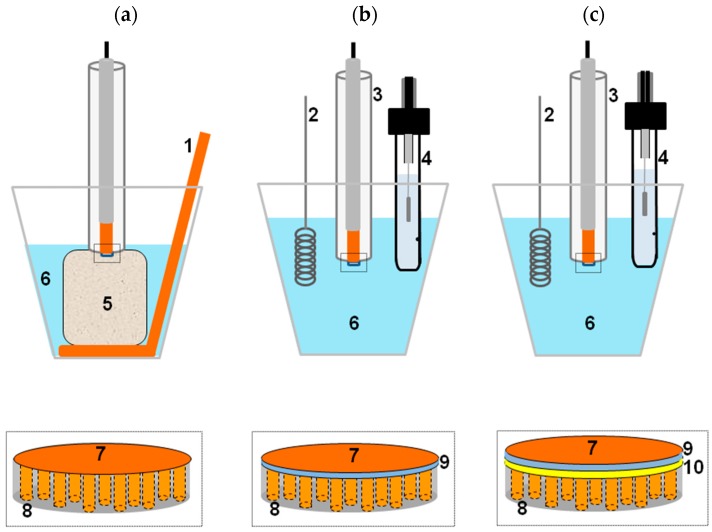
Scheme illustrating three template deposition methods used which differ for the way used to contact the PC membrane (8) to the flat disk Cu electrode (7). From left to right: (**a**) adhesion by the pressure furnished by the melamine foam (5); (**b**) adhesion by using a Nafion interlayer (9) as polyelectrolytic glue; (**c**) as (**b**), but using a PC membrane pre-sputtered with the gold interlayer (10). Other components: (1) Cu counter electrode; (2) Pt counter electrode; (3) working electrode; (4) Ag/AgCl KCl sat reference electrode; (6) 0.4 M CuSO_4_, 0.01 M H_2_SO_4_ solution (reprinted with permission from [29]).

To perform the electrochemical deposition inside the pores of a nanoporous membrane it is necessary that one side of the membrane be used as the working electrode. This can be achieved by plasma or vacuum deposition of a thin layer of metal (typically 100–200 nm) on one side of the membrane, or placing the membrane in intimate contact with a solid electrode. Figure 2 shows three possible modes for template electrochemical deposition [28,29]: in Figure 2a, the membrane is pressed onto a solid electrode by a sponge drenched in the electrolyte [30], in Figure 2b,c adhesion between the membrane and underlying electrode is achieved thanks to a Nafion interlayer used as polyelectrolytic glue. The inner face of the membrane can be bare or pre-sputtered with a gold thin-layer, to improve electrical contact (Figure 2b,c, respectively). In electrochemical template deposition, the coated film is placed in an electrochemical cell, acting as the cathode and a counter electrode is the anode.

**Figure 3:**
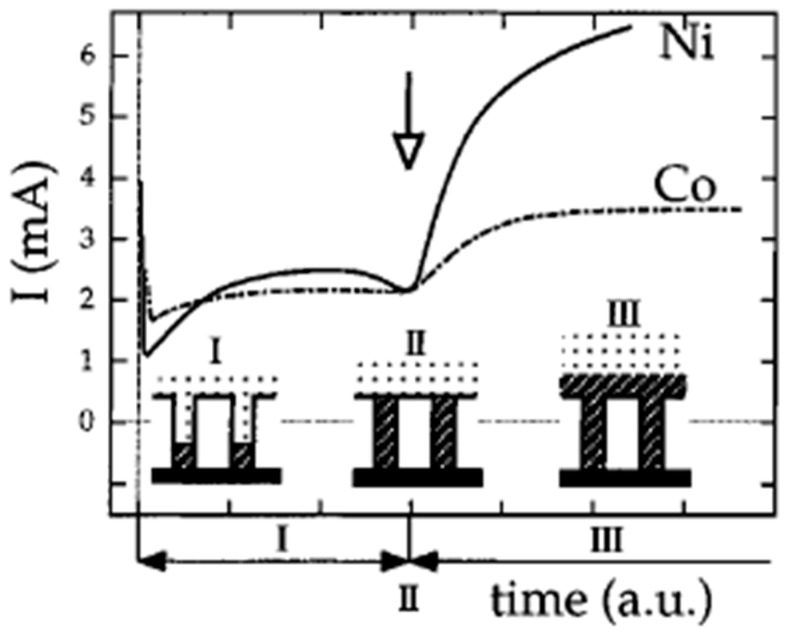
Electrochemical reduction current as a function of time for the potentiostatic platin of Ni and Co in the pores of PC membrane with 80 nm of nominal diameter (reprinted with permission from [24]).

The deposition can be performed under potentiostatic or galvanostatic conditions. In the former case, it is possible to monitor the time course of the deposition and the progressive filling of the pores by analyzing the time transient current which is characterized by a sigmoidal shape [24,28,31] (Figure 3). The initial current is characterized by an intense peak and a fast decay due to the depletion of metal ions following the fast initial deposition and the increase of resistance inside the pores of the membrane. In phase I the current reaches a plateau which corresponds to the progressive filling of the pores. In phase II, the current increases again because of the increase of the electrode area caused by the growing of the metal outside the pores. In this phase it is possible to observe caps on the tips of the nanowires with a typical mushroom shape [24]. Finally, the overgrown caps merge together producing an almost flat surface; this leads to a second plateau in the current transient (phase III). If the goal is the preparation of ensembles of nanodisk or nanowire electrodes, it is essential to stop the electrodeposition at the end of stage I, i.e., before the “mushroom caps” start to grow.

The electrodeposition of metals has been applied in order to obtain nanowires not only of gold, but also of other materials, such as, other metals (Co [24,32,33], Ni [24,28,34], Cu [24,28,29], Pt and Pd [35]), alloys (NiFe [33], FeSiB [34]) or salts (Bi_2_Te_3_ [36], CdS [23]). Some recent papers deal with the theoretical modeling of electrochemical deposition in template membranes [37,38,39,40,41,42]. 

### 2.2. Template Electroless Deposition 

The electroless deposition involves the chemical reduction of a metal salt from the solution to an activated surface. Activation is performed by generating metal nuclei on the surface of a non-catalytic material. In the following chemical reaction, the metal ion is reduced more quickly at the sensitized surface so that this surface is rapidly plated with the desired metal [43]. 

The electroless deposition of gold in template membranes involves four steps [1,20,44]: (i) “sensitization” of the membrane, adsorbing Sn^2+^ ions on the substrate; (ii) deposition of Ag nanoparticles by reduction of Ag^+^ by the adsorbed Sn^2+^ ions; (iii) galvanic displacement of the Ag particles by reduction of a Au(I) solution; (iv) catalytic reduction of more gold on the deposited Au nuclei by addition of a reducing agent (formaldehyde). Note that this procedure applies to PC template membranes made hydrophilic by impregnation with the wetting agent polyvinylpyrrolidone, as usually done by track-etch membrane providers.

In contrast with the electrochemical template deposition, in the electroless method the metal layer grows from the catalytic nuclei, which are located on the pore walls, towards the center of the pores. When step (iv) is stopped at short times (e.g., 40–60 min at pH 10 [26]) one can obtain hollow tubes instead of nanowires [45,46] which can be further functionalized, for instance with the well-known thiol chemistry [47], for application as molecular sieves [48].

Other metals, such as Cu [49], Pd [50] and Ni-P [51] can also be deposited in polycarbonate templates by electroless deposition. In this case a suitable procedure for the desired metal might be applied.

**Figure 4:**
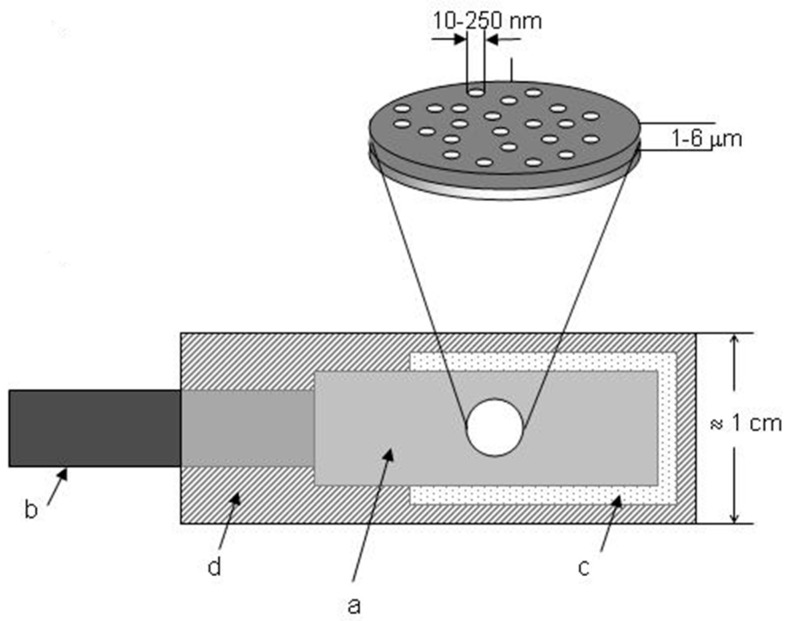
Schematic representation of a NEE, prepared by using a track-etched polycarbonate membrane as template: (a) track-etched golden membrane; (b) copper adhesive tape with conductive glue to connect to instrumentation; (c) aluminium adhesive foil with nonconductive glue; (d) thermoadhesive insulating tape (e.g., Monokote by Topflite). Note: the dimensions of the pores (nanofibres) are only indicative and not to scale (reprinted with permission from [52]).

Figure 4 shows the final structure of an ensemble of nanoelectrodes to be used as an electrode easy to handle [52]. The metalized membrane is sealed with an insulating film (d) in which a circular hole is punched, which defines the geometric area of the NEE (A_geom_), that is the overall surface of the ensemble (nanoelectrodes and insulator between them) exposed to the solution. As a final step, the NEE assembly is heat-treated at 150 °C for 15 min to produce a water-tight seal between the gold nanowires and the surrounding polycarbonate.

Note that A_geom_ can be changed at pleasure [53,54] without influencing the signal/noise (S/N) ratio which is typical of NEEs. The copper tape (b) is used for electrical connection with the potentiostat. 

### 2.3. Combined Electroless-Electrochemical Deposition

**Figure 5:**
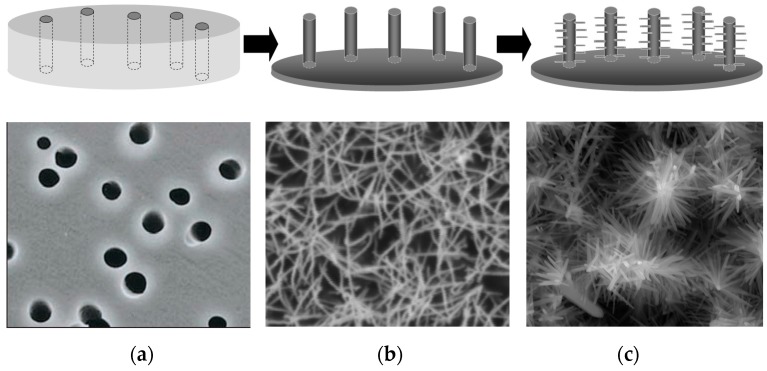
Scheme illustrating the preparation procedure used to obtain Au nanowires decorated with hierarchically branched ZnO. (**a**) scheme and SEM image of the template membrane; (**b**) scheme and SEM image of an ensemble of gold nanowires obtained by electroless deposition; (**c**) scheme and SEM image of ZnO nanostructures grown electrochemically on the Au nanowires (reprinted with permission from [55]).

It was recently demonstrated that ensembles of Au nanowires prepared by template electroless deposition can be decorated electrochemically with metal and oxide nanostructures to obtain functional nanoarrays with special electrocatalytic or photocatalytic properties. By using electrochemically initiated oxide deposition [55] it was possible to prepare hierarchically branched ZnO nanostructures on Au nanowires, such as those shown in Figure 5. 

Very recently, it was demonstrated that closed bipolar electrochemistry can be successfully applied for the asymmetrical deposition of Cu_2_O and TiO_2_ on the two opposite tips of electroless templated Au nanowires [56]. Note that with this approach, the PC template membrane was kept in place and acted as separator between the two half-cells in the closed bipolar cell configuration. This geometry allowed to perform the bipolar deposition by applying a lower potential than the one required when operating with an open bipolar cell architecture.

## 3. Diffusion at Arrays or Ensembles of Nanoelectrodes

At the dimension range typical of nanoelectrodes (tens of nm) edge effects are predominant and the diffusion from the bulk of the solution to individual nanoelectrodes is described by a radial geometry [57].

For instance, for a spherical microelectrode, the thickness, δ(t), of the diffusion layer around the electrode is given by Equation (1) [57]:

1/δ(t) = [1/(πDt)^1/2^] + 1/r (1)

where D is the diffusion coefficient of the species, t is the time scale of the experiment, and r is the radius of the electrode. As the electrode decreases in size, the diffusion layer thickness approaches the size of the electrode dimension. The steady-state diffusion-controlled limiting current, I (t→∞), is proportional to the inverse of the diffusion layer thickness, according to Equation (2) [57]:

I (t→∞) = nFAC°/δ(t→∞)
(2)

where n is the number of electrons exchanged, F is faraday constant, A is the electrode surface area, and C° is the bulk concentration of the redox species. Dividing Equation (2) by A, it is evidenced that the smaller (nano)electrodes will provide higher current densities as a consequence of this enhanced mass transport component.

When the thickness of the diffusion layer is so small as to be comparable with the thickness of the electrical double layer (a few tens of nm), electrostatic forces between the ions in the double layer and the redox analyte can accelerate (or retard) the flux of redox species with ionic charge opposite (or equal) to the ions in the double layer, so generating the conditions for a further enhancement (or lowering) of the mass transport to the nanoelectrode surface. Dickinson and Compton [58] presented a first attempt to analyze these effects, providing numerical solutions of the Poisson-Boltzman equation, calculated for hemispherical nanoelectrodes of vanishing size. Their study revealed significant effects of curvature on the diffuse double layer profiles, which become relevant for electrodes with radii less than 50 nm, even in the presence of supporting electrolyte. An enhanced driving force is therefore expected for nanoelectrodes as compared to electrodes larger than 50–100 nm [58]. Studies on the kinetics of electron transfer at individual nanoelectrodes has been reviewed by Chen and Liu, with particular focus on the role of electrostatic interaction within the electrical double layer, and overlap between the diffusion layer and the electrical double layer [59]. Further studies have analyzed mass-transport processes at nanoelectrodes and their arrays [60]. 

**Figure 6:**
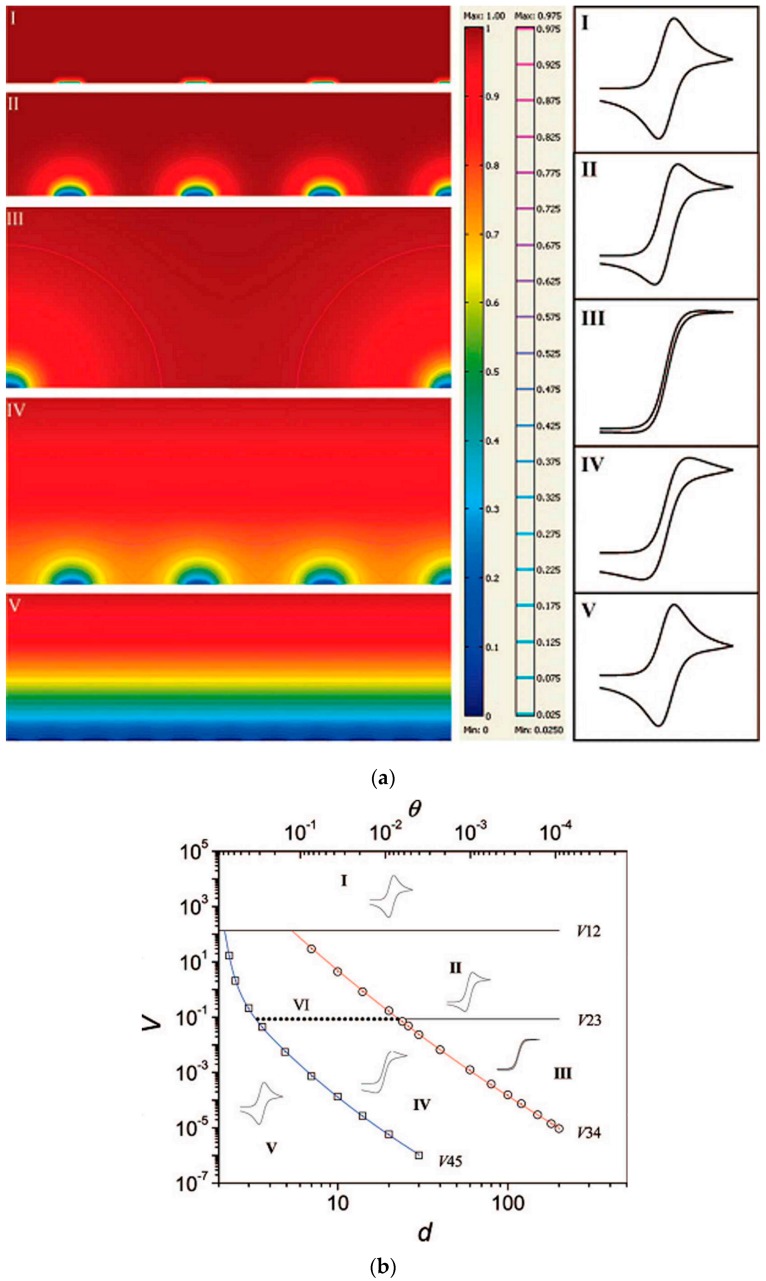
(**a**) Simulated concentration profiles and relevant voltammetric patterns, for microelectrode arrays representing the five main categories of possible diffusion modes (from I to V). In the scale bar, the red and blue colour represents the bulk concentration and zero concentration, respectively. The second scale bar represents a relative concentration scale for the contour lines. (**b**) Zone diagram of cyclic voltammetric behaviour at electrode arrays: d is the centre-to-centre distance of individual electrodes in the array (measured in units of a), V is the dimensionless scan rate, and θ is the fraction of electrochemically active area in the array (reprinted with permission from [64]).

Taking into account the overall diffusive process for a whole array of nanoelectrodes, it is evident that NEEs/NEAs can exhibit different voltammetric responses depending on the scan rate or the reciprocal distance among the nanoelectrodes [61,62,63]. The different limit situations are summarized in Figure 6a. When radial diffusion boundary layers totally overlap, i.e., when the diffusion hemisphere is larger than the mean hemidistance among the nanoelectrodes, NEEs behave as macroelectrodes with respect to the Faradic current (total overlap regime, peak shape voltammograms, case V). When the diffusion hemisphere becomes shorter (higher scan rates) or the hemidistance among nanodisks is larger, the voltammetric response is dominated by radial diffusion conditions at each element (pure radial regime, sigmoidally shaped voltammograms, case III). At very high scan rates, the linear active state is reached (case I), where linear diffusion is predominant at each nanodisk (peak shaped voltammograms, but with peak currents much smaller than case V). Obviously, intermediate situations can be also observed (case IV and II).

Theoretical studies [61,64,65,66,67] have examined in detail the role of the different diffusion regimes on the voltammetric responses recorded at arrays of ultramicro- and nanoelectrodes. In particular, Guo and Lindner [64] introduced a very useful zone-diagram where the combination of suitable dimensionless parameters allows one to determine the diffusion regime (and kind of voltammetric response) operative for a specific geometry of the array, at a specific voltammetric scan rate (see Figure 6b). The study was focused on arrays of microelectrodes, but can be extended to arrays of nanoelectrodes. Note that such a simulation was developed for arrays in which the effects at the border of the array are negligible, that is for arrays including a very large number of electrodes [53,61,68]. This condition can be achieved for small size arrays only if the electrodes size is very small, i.e., at nanoscale level. To make border effects negligible it is necessary to increase significantly the overall number of electrodes in the array; for instance in a 10^4^ electrodes squared array only 3.96% of them will be on the perimeter, while in a 100 electrodes array, 36% of the electrodes experience border effects.

A distance between the electrodes of 10r (where r is the radius of the individual electrodes) is high enough to avoid cross-talking between the electrodes [61]. This means that if r = 10 µm, the side of a 10^4^ electrodes-array will be as large as 1 cm. On the other hand, if r = 0.1 µm, the side of the array, (with the same number of electrodes), will be lowered to 100 µm. This is particularly important dealing with electrochemical biosensors, where the immobilization of expensive biomolecules on the surface of the electrode is needed and miniaturization is a must.

On the other hand, in the case of arrays composed by a small number of nanoelectrodes, border effects play a relevant role. Under these conditions, when the overall size of the array is in the µm range, even for arrays operating in total overlap condition, sigmoidally shaped voltammograms are observed [7].

A recent paper presented quantitative theoretical investigation for development and validation of an analytical model for prediction of chronoamperometric responses at random arrays of micro- and nanodisk electrodes. This model was based on a 3D Brownian motion approach; it allowed to propose a simple analytical equation useful to predict the chronoamperometric behaviour of commonly used irregular arrays [69]. 

## 4. Voltammetry with NEEs and NEAs

### 4.1. Voltammetry with NEEs

The diffusion regime usually observed at NEEs fabricated from commercially available track-etched membrane is the total overlap regime [20], nevertheless, transition from this regime as a function of the nanoelements distance has been experimentally demonstrated using specially-made membranes [62] or by increasing the electrolyte viscosity [70]. 

For NEEs operating under total overlap diffusion conditions the Faradaic current (I_F_) is proportional to the total geometric area of the ensemble exposed to the sample solution (A_geom_, area of the nanodisks plus the insulator area), while the double layer capacitive current (I_C_), which is the main component of the noise in electroanalytical chemistry, is proportional only to the active area (A_act_), that is the area of the metal nanoelectrodes exposed to the electrolyte [20].

The geometric area of a NEE is defined by the dimension of the hole punched into the insulator (see Figure 4). The active area is calculated from the membrane characteristics such as, pore density (q) and pore radius (r), according to:

A_act_ = π·r^2^·q·A_geom_ (3)

The ratio between the active and the geometric area defines a key parameter named fractional electrode area (f):

f = A_act_/A_geom_ (4)

Faradaic-to-capacitive current ratios at NEEs and conventional electrodes with the same geometric area are related by Equation (5) [71]:

(I_F_/I_C_)_NEE_ = (I_F_/I_C_)_conv_·f (5)

Equation (5) explains why detection limits (DLs) at NEEs can be 2–3 orders of magnitude lower than with conventional electrodes [20,71,72,73]. Since the improvement in S/N ratios are strictly related to the fractional area, the electroanalytical performances of NEEs are not affected by any variation in the geometric area as long as the active area changes accordingly, i.e., the f parameter is kept constant [53,54].

Some diagnostic criteria can be given to distinguish “good” NEEs from “bad” NEEs [44,52]. Voltammograms affected by a large capacitive current are indicative of poor sealing between the nanowires and the surrounding PC insulator and/or by heavy scratching of the PC membrane caused by an improper handling of the NEE. On the other hand, a radial diffusive contribution to the overall signal suggests a larger distance between the nanoelectrodes, possibly due to an only partial filling of the pores of the template.

### 4.2. Ordered Arrays of Nanoelectrodes Fabricated by Nanolithography

Top down techniques, such as ion beam lithography [3,74,75], electron beam lithography (EBL) [76], nanoimprint [77] or scanning probe lithography [78,79] allow one to achieve high resolution nanostructuring, providing a precise positioning and sizing down to a scale of a few nanometers. This spatial resolution capabilities have been indeed exploited to prepare ordered arrays of nanoelectrodes [3,7,76]. 

Studies on arrays of gold nanolectrodes [68] demonstrated that PC can be used as an high resolution resist for e-beam lithography with the advantage of low cost and suitability for easy chemical functionalization with biomolecules [9,80]. 

**Figure 7:**
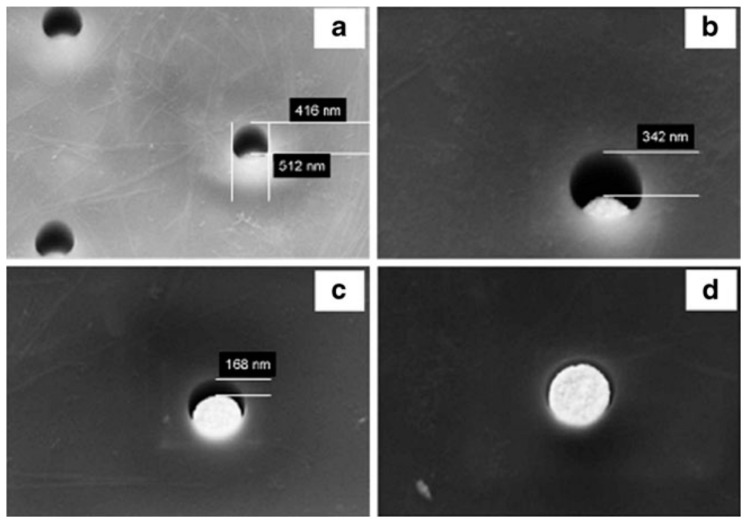
SEM images of NEAs with holes 500 nm in diameter with gold electrochemically deposited inside for 0 s (**a**); 10 s (**b**); 20 s (**c**); and 30 s (**d**). Estimated recession depths: (**a**) 450 nm; (**b**) 300 nm; (**c**) 150 nm; (**d**) 0 nm (reprinted with permission from [68]).

PC based nanoelectrodes are indeed fabricated by patterning arrays of holes in a thin film of PC spin-coated on a gold layer on Si/Si_3_N_4_ substrate. The PC surface is exposed to the e-beam and the tracks developed (etched) in KOH [68]. These holes can be used as openings for recessed nanoelectrodes, however, by further electrochemical deposition of gold, it is possible to fill partially or totally the holes up to obtain arrays of inlaid nanodisk electrodes (Figure 7). The perfect control of the geometry of the array and recession degree allows the full control of the diffusion regime at the so obtained NEA [68].

**Figure 8:**
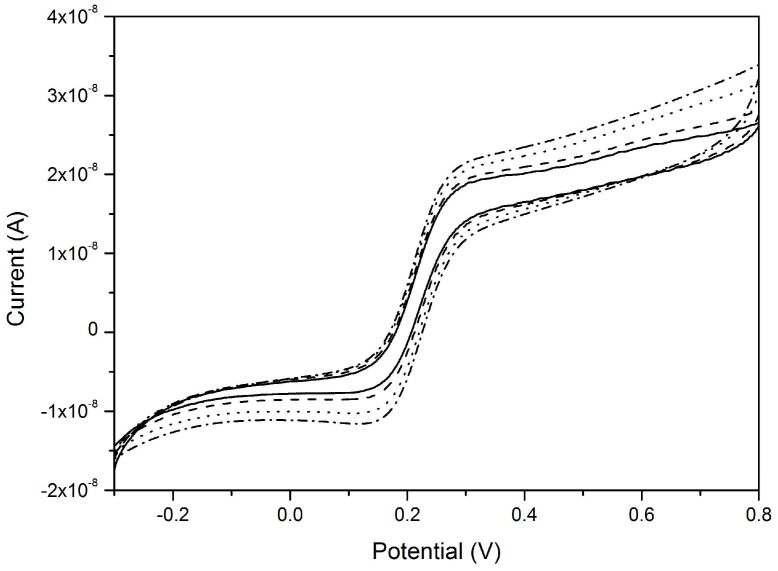
CVs recorded at a gold nanoelectrodes array in 10^−4^ M ferrocene methanol and 0.5 M NaNO_3_. Scan rates: 5 (full line), 10 (dashed line), 20 (dotted line), and 50 mV·s^−1^ (dash–dot line). Geometrical characteristics: nanodisk radius = 75 nm, distance centre-to-centre = 3 μm, number of nanoelectrodes in the array = 1.1 × 10^4^ (reprinted with permission from [68]).

Figure 8 demonstrates the scan rate independency of voltammetric signals at a NEA operating under pure-radial diffusive control; the geometric characteristics of the NEA and experimental conditions are indicated in the figure caption. Figure 8 refers to the case of an array of inlaid nanoelectrodes; it is worth to stress that, because of the nanolithographic process itself, quite often the nanoelectrodes so obtained can be slightly recessed, so that theoretical model for such geometries must be eventually taken into account [68,75].

**Figure 9:**
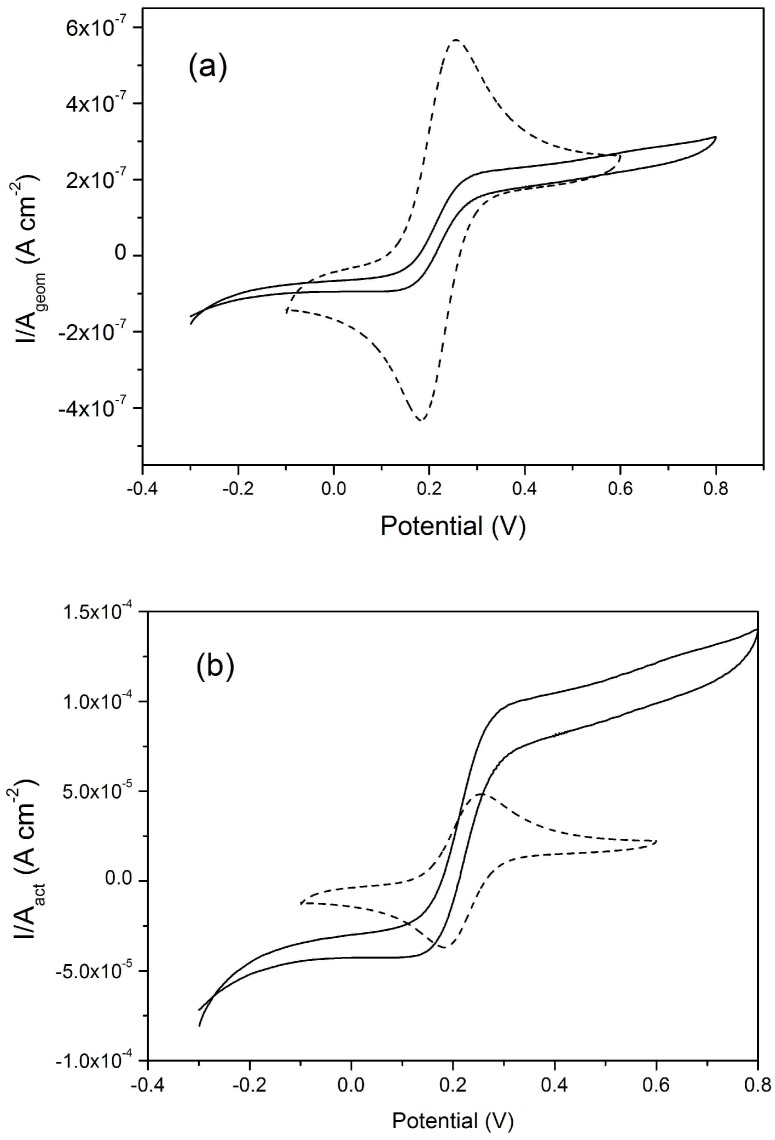
CVs recorded at a NEA (full line, see Figure 8) and at a NEE (dashed line, nanoelectrode radius 15 nm, A_geom_ = 0.07 cm^2^, A_act_= 4.5 × 10^−3^ cm^2^) made of Au-nanodisks in polycarbonate, plotted using current densities calculated respect to the geometric area (**a**) and active area (**b**); scan rate 10 mV·s^−1^, in 10^−4^ M ferrocenemethanol. For further details, see the original article (reprinted with permission from [68]).

The availability of NEEs (operating under totoal overlap diffusion conditions) and NEAs (operating under pure radial diffusion conditons), both fabricated with the same materials (namely, gold nanoelectrodes embedded in PC) has made it possible to perform the direct comparison between these two diffusion regimes in terms of current density yields. Note that with arrays and ensembles, the current density can be calculated in two ways, since the measured current can be normalized with respect to: (i) the active area of the array; (ii) the overall geometric area. The first mode, quantifies indeed the current density at each individual nanoelectrode while the latter defines the same parameter with respect to the array as a whole. Figure 9, taken from Moretto et al. [68], compares the current densities for a NEE and NEA made of Au-nanodiscs in PC, operating at 10 mV·s^−1^ under total overlap and pure radial diffusion conditions, respectively. In Figure 9a, the current density is calculated with respect to the geometric area and in Figure 9b with respect to the active area. 

These plots show that the current density at the NEE is higher than the one at the NEA when the overall geometric area of the array is taken into account. On the other hand, this ratio is fully reversed in favour of the NEA (under pure radial diffusive control) if one evaluates the current density with respect to the active area. This is because at the NEE in total overlap condition, 100% of the geometric area contributes to produce the Faradic signal, while at the NEA under pure radial condition, the nanoelectrodes do not cross-talk and the transduction efficiency at each nanoelectrode reaches its maximum. Studies on arrays of gold nanolectrodes [68] demonstrated that PC can be used as an high resolution resist for e-beam lithography with the advantage of low cost and suitability for easy chemical functionalization with biomolecules [9,80]. 

### 4.3. Nanoelectrode Arrays of Boron-Doped Diamond

A novel material which is increasingly used for the preparation of NEAs is boron-doped diamond (BDD). BDD is indeed a very attractive electrode material for the fabrication of nanostructures thanks to its excellent chemical and electrochemical stability, satisfactory electrical conductivity and wide potential window accessible [81]. The preparation of arrays and ensembles of BDD nanoelectrodes have been achieved by nanoparticles template procedure and nanosphere lithography [82,83], by e-beam lithography [84] and by focused ion-beam milling [85]. BDD-NEAs have been characterized by scanning electrochemical microscopy (SECM) [86] and applied to study, at the nanoscale, relevant interfacial processes, such as adsorption phenomena at hydrogen- and oxygen- terminated BDD nano-surfaces [87] or to visualize ion-transfer across nano-interfaces between two immiscible electrolyte solutions [85]. 

**Figure 10:**
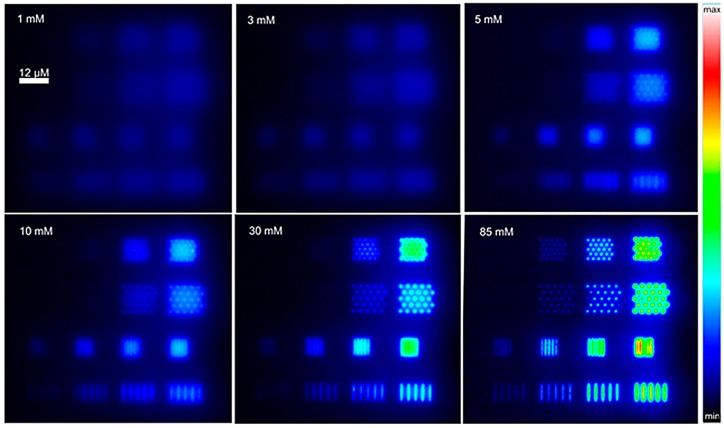
ECL images of a BDD-NEA obtained in phosphate buffer (pH 7.4) containing 1 mM Ru(bpy)_3_^2+^ and increasing concentrations of TPrA (indicated in top-left corner of each box). Images were recorded in the dark with a ×50 objective when applying a constant potential of 1.2 V vs. Ag/AgCl/KCl. All images were coded according to the same false colour scale (right) (reprinted with permission from [88]).

It was recently shown that e-beam nano-lithographed BDD-NEAs can be used to finally tune electrochemically induced luminescence (ECL) as a function of the array geometry and/or composition of the electrolyte solution containing the luminophore [Ru(bpy)]_3_^2+^ and the co-reactant, tri-n-propylamine (TPrA) [88], as illustrated in Figure 10. In particular, NEAs with 16 different geometries were fabricated on the same BDD substrate, allowing one to detect simultaneously ECL emission from the different NEAs. The analysis of the ECL imaging data indicated that the ECL emitting zone scales inversely with the co-reactant concentration as well as significantly more intense ECL signals were detected for NEAs operating under overlap conditions.

These plots show that the current density at the NEE is higher than the one at the NEA when the overall geometric area of the array is taken into account. On the other hand, this ratio is fully reversed in favour of the NEA (under pure radial diffusive control) if one evaluates the current density with respect to the active area. This is because at the NEE in total overlap condition, 100% of the geometric area contributes to produce the Faradic signal, while at the NEA under pure radial condition, the nanoelectrodes do not cross-talk and the transduction efficiency at each nanoelectrode reaches its maximum. 

### 4.4. Electron Transfer Kinetics

NEEs/NEAs are very sensitive to the electron transfer kinetics [20]. According to model proposed by Amatore et al. [89], as well as to more recent theoretical models [64,65,66], a NEE behaves as a partially blocked electrode (PBE), whose current response is identical to that of a naked electrode of the same overall geometric area, but with a smaller apparent rate constant (k°_app_) for the electron transfer, which decreases as the coverage of the surface increases. According to this model, the nanodisks electrodes are the unblocked surface and the template membrane is the blocking material. The apparent rate constant (k°_app_) is related to the true standard rate constant by the following equation:

k°_app_ = k° (1 − ϑ) = k° f (6)

where ϑ = (A_geom_ − A_act_)/A_geom_ and f is the fractional electrode area (see Equation (4)).

From an analytical viewpoint, the operativity of Equation (6) means that high faradaic peak currents are observed at NEEs only for redox couples with “very reversible” behavior. In cyclic voltammetry (CV) in fact, the reversibility of a redox system depends on the k° value and on the scan rate (v). Using conventional electrodes, reversible patterns are obtained when:

v^1/2^ ≤ (k°/0.3) (7)

but if NEEs are used, k° is substituted by k°_app_, and the previous relation becomes:

v^1/2^ ≤ [(k° f)/0.3] (8)

Considering that mean f values ranges from 10^−2^ to 10^−3^, from Equation (8) we can conclude that the scan rate value that defines the transition between reversible and quasi-reversible behavior is placed at a scan rate 2–3 orders of magnitude lower than the value requested for conventional electrodes. This is a limitation to be seriously taken into account when trying to optimize NEEs for bioanalytical application, since it is important to consider the contrasting effect both of the increased I_F_/I_C_ value and the apparent slowing down of the electron transfer kinetics. On the other hand, from a mechanistic viewpoint, it is an advantage since it means that with NEEs it is easier to measure experimentally very large k° values [57]. By the analysis of ΔE_p_ dependence on the scan rate [90] and using suitable working curves [91], smaller k°_app_ values are obtained and converted to larger k° by Equation (6) [89].

## 5. From 2D- to 3D-NEAs 

**Figure 11:**
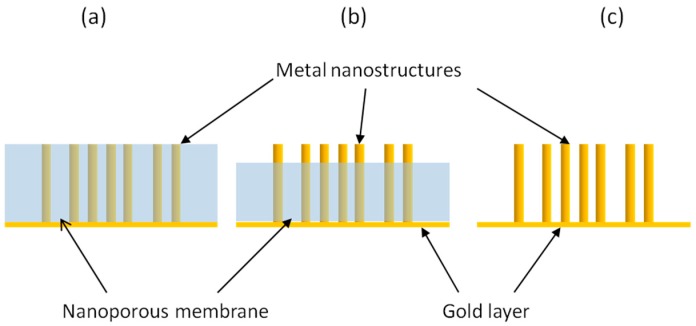
Different geometries for template nanoelectrode ensembles: (**a**) ensemble of nanodisks; (**b**) ensemble of partially naked nanowires; (**c**) ensemble of completely naked.

Depending on the final surface morphology of the NEE, one can prepare two-dimensional nanoelectrode ensembles (2D-NEEs), made of ensembles of nanodisk electrodes, or three-dimensional nanoelectrode ensembles (3D-NEEs), made of nanofibers [92,93,94,95]. The small surface area of the NEEs can be increased in a controlled way by suitable etching, in order to partially remove the upper layers of the polycarbonate template membrane. In other words, NEEs of metal nanodisks, prepared by electroless or electrochemical template deposition within the pores of track-etched polycarbonate membranes, are treated with oxygen plasma [92] or with solvent mixtures such as CH_2_Cl_2_/C_2_H_5_OH mixtures [5,96], to achieve the controlled etching of the template. This causes the structure of the final ensemble to change from a 2D flat structure into a 3D one [5]. As illustrated in Figure 11, depending on whether the template is kept on site, partially etched or fully removed, it is possible to obtain nanoelectrodes ensembles with very different geometries. 3D-NEEs are powerful electrode systems with a high active surface suitable for functionalization and extreme miniaturization. For 3D-NEEs the measurement of the active area is necessary to correlate the intensity of electrochemical signals with A_act_ values. This parameter can be obtained by AC electrochemical impedance spectroscopy [97] or by measuring: the double layer charging current [29] or the total charge associated to gold oxide stripping [98] or to other redox processes relevant to adsorbed species, such as polyoxometallates [5].

In order to take maximum advantage of the use of 3D-NEEs, one has to take into account that their electrochemical response is influenced by the high density of metal nanofibers (~6 × 10^8^ nanofibers cm^−2^), and possible overlap of the diffusion layers [99]. The parameters which rule the electrochemical behavior of 3D-NEEs were studied by De Leo and coworkers [5]. In this work it was shown that for fast redox couples such as ferrocene derivatives, faradaic peak currents are not influenced by the etching process, while double-layer capacitive currents increase. Since 3D-NEEs behave as PBE, the true kinetic constant is indeed substituted by an ‘apparent kinetic constant’ (see Equation (6)). However, for a very fast redox couple, the influence of the change of *k*°_app_ values by changing f cannot be appreciated experimentally at the scan rates typically used for cyclic voltammetry (100 mV·s^−1^ or lower). As predicted on the basis of the theoretical model by Amatore et al. [89], the situation changes dramatically for redox species characterized by slow heterogeneous electron transfer kinetics.

**Figure 12:**
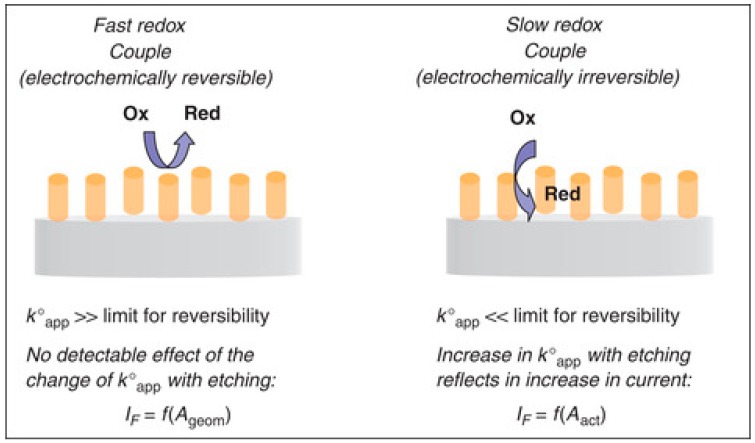
Sketch of the effect of different electron transfer kinetics on electrochemical responses at three-dimensional nanoelectrode ensemble (3D-NEE) (reprinted with permission from [101]).

The kinetic limitation causes a considerable torsion of the lines of flux near each nanoelectrode element, thus imposing a local rate of diffusion considerably larger than the one far from each electrode. As a consequence each nanoelectrode behaves individually with respect to the heterogeneous kinetics. Figure 12 shows a sketch summarizing the effect of differences in heterogeneous rate constants on the electrochemical behavior of 3D-NEEs. Recently, a general theoretical treatment of the electrochemical behavior of arrays with 2D and 3D geometries have been proposed by Amatore and coworkers [100]. 

## 6. Bio-Analytical Applications of NEEs/NEAs: First Studies

The improved signal-to-noise (S/N) ratio and extreme miniaturization typical of 2D-NEEs, have made them particularly attractive for analytical applications to detect electroactive species at low concentration levels. First studies performed at the end of the 1990s−beginning 2000s have proven that NEEs are powerful electroanalytical tools for the analysis of small redox molecules with reversible electrochemical behavior, such as ferrocene derivatives, ruthenium complexes, phenothiazines or viologens [20,72,73]. It was demonstrated that NEEs can be used for determining iodide anions in ophthalmic drugs [44] or in natural waters [102]. Moreover, they can be successfully applied also for the direct detection of trace levels of the complex redox bio-macromolecules such as the heme-protein cytochrome *c* [53,103]. The application of NEEs has been studied more recently to perform the analysis of heavy metals and toxic elements at trace and ultra-trace concentration levels. Mardegan et al. demonstrated the applicability of NEEs to perform trace analysis of arsenic and its redox speciation by anodic stripping-square wave voltammetry (AS-SWV) [104]. For analyzing heavy metal ions, NEEs can be modified by deposition of a Bi film, so extending the cathodic limit of the accessible potential window, thanks to the high overpotential of Bi for the hydrogen evolution reaction. By this way it was possible to apply NEEs for the anodic striping voltammetric determination of trace levels of lead(II) [105]. 

In other cases, it is convenient to exploit for analytical purposes the larger surface area of 3D-NEEs. For example, Stortini et al. demonstrated efficient sensing performances of copper 3D-NEEs with respect to nitrate determination at concentration levels as low as few µM [29]. Similarly, Cao et al. demonstrated that 3D-NEEs composed of gold nanowires can be successfully applied to the analysis of the antibiotic daunorubicin [98].

The small active area of 2D-NEEs can be a limit also when the gold nanodisks are used as substrate for the immobilization of bioactive molecules, as typically done for preparing electrochemical biosensors; also in these cases the use of 3D-NEEs is advisable. The gold surface of 3D-NEEs have been used for the determination of ovarian cancer marker mucin-16 (MUC16) by Viswanathan et al. [106]. These authors developed an electrochemical immunosensor using monoclonal anti-mucin-16 antibodies (αMUC-16) bonded on liposomes in which ferrocene carboxylic acid is encapsulated; αMUC-16 was also immobilized on a cysteamine self-assembled monolayer (SAM) on the gold surface of 3D-NEE via cross-linking with carbodiimide (EDC) and N-hydroxysulfosuccinimide (Sulfo-NHS). A sandwich immunoassay was performed on αMUC-16 functionalized 3D-NEE with MUC-16 and immunoliposomes using differential pulse voltammetry (DPV) as the detection technique. This example shows the possibility to exploit the increased Au surface of 3D-NEEs to increase the amount of adsorbed biomolecules, however such an improved surface area comes at the expense of an increased capacitive current. This is not a major problem for the direct detection of molecules adsorbed on the nanowires, since both the amount of redox species and the capacitive current scale proportionally with the active area. On the other hand, when the detection involves a diffusion step (of the analyte, mediator or substrate) the etching can cause a significant decrease of the S/N ratio, as described in Section 5. In order to overcome such a drawback, Ugo and coworkers proposed an original approach in which the biorecognition element is immobilized on the polymeric template of the NEE. In such a design, the transducer and the biorecognition elements are not overlapped but integrated in strict proximity. This approach, besides maintaining the excellent detection limits of 2D-NEEs, greatly increases the amount of biomolecules bound on the NEE, since the polycarbonate surface is 2–3 orders of magnitude larger than the gold surface of the nanoelectrodes. 

**Figure 13:**
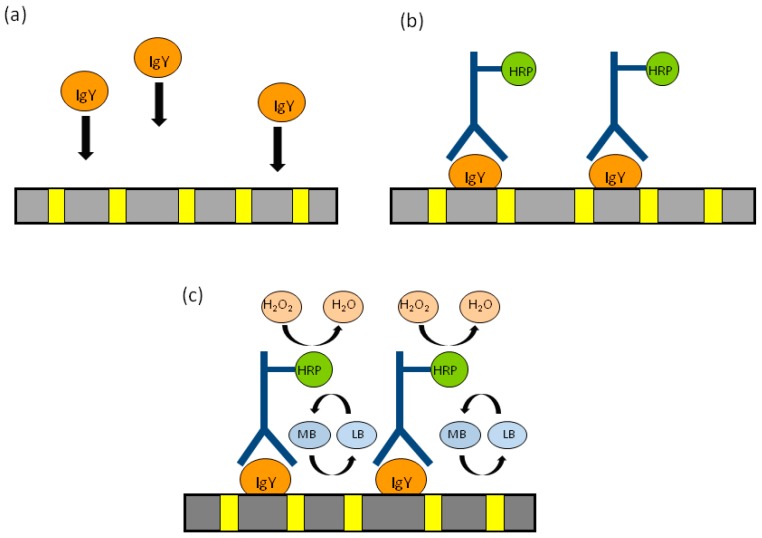
Schematic representation of the analytical protocol used to detect immunoglobulin IgY, extracted from tempera paints. MB and LB are the oxidized and reduced forms of the redox mediator methylene blue and HRP is horseradish peroxidase, used as enzymatic label for the anti-IgY antibody (**a**) capture of immunoglobulin IgY on the polycarbonate of the NEE; (**b**) molecular recognition by anti-IgY labeled with HRP; (**c**) generation of the electrocatalytic cycle by adding the enzyme substrate H_2_O_2_ and the mediator MB. (reprinted with permission from [109]).

This can be done by exploiting the functional groups inherently present in the template matrix. Indeed, titrations with thionin acetate indicated that a surface concentration of -COOH in the order of 9.7 × 10^−10^ mol·cm^−2^ is naturally present on the surface of track-etched polycarbonate; this number can be increased to 3.4 × 10^−9^ mol·cm^−2^ by controlled oxidation with KMnO_4_ [107,108]. Mucelli et al. proposed this strategy for the preparation of an immunosensor for the determination of the HER2 receptor, overexpressed in certain kinds of breast cancer [9]. At first, the specific antibody trastuzumab is immobilized on the PC of a NEE. Later on, it is incubated with the sample to capture the target protein HER2. The captured protein is then reacted with a different primary antibody (namely, monoclonal CB-11) which finally binds a secondary antibody labeled with horseradish peroxidase (HRP). The electrochemical signal is generated by methylene blue (MB) added to the solution as redox mediator, which shuttles electrons from the nanoelectrode elements to the HRP, when the latter reacts with its substrate, i.e., H_2_O_2_. A similar approach has been used for determining the presence of egg yolk as binder in ancient tempera paintings [109]. The detected analyte was the glycoprotein immunoglobulin IgY which is the main immunoglobulin in chicken eggs. In this approach, which is schematized in Figure 13, IgY is captured by the polycarbonate surface (A), the electrode is then incubated with anti-IgY labeled with HRP (Anti-IgY-HRP) (B) and, finally, the presence of the label HRP, is detected by adding the enzyme substrate H_2_O_2_ and a mediator (C). The main advantage of this approach lies on the fact that the antigen is bound directly on the polycarbonate surface, reducing the analytical steps and the reagents required. 

Also other molecules in the sample can spontaneously bind to the polycarbonate, however only IgY is recognized by the HRP-labeled anti IgY, which finally generates the electrocatalytic signal. This allowed to develop a diagnostic scheme able to distinguish qualitatively, but with high precision, egg-yolk tempera (which contains IgY) from other kind of tempera (not containing IgY) as well as oil or acrylic paints. It is worth pointing out that the application of this geometry for the sensitive detection of DNA hybridization was also demonstrated using 2D-NEEs or NEEs decorated with Au-nanoparticles [107]. 

## 7. Most Recent Advances in Biosensing with NEEs/NEAs

In this section we highlight very recent advances dealing with the use of nanoelectrode arrays and ensembles as sensing platforms suitable for therapeutic, diagnostic and bioanalytical application. In Table 1, Table 2 and Table 3 are listed and briefly commented examples of researches in this field published in the last five years. The nanoelectrode arrangements studied include both NEEs and NEAs structures composed by nanodisks, nanowires, nanochannels, nanopores and nanotubes. These examples demonstrate the variety of geometries and morphologies of nanoelectrode structures that can be successfully applied for biosensing purposes, offering excellent detection capabilities for analyzing and monitoring different biomarkers and proteins, DNA, neurotransmitters, metabolites, pharmaceutical or toxic compounds. 

Concerning the direct detection of small molecules (see Table 1) many recent researches focused on the use of 3D-arrays of nanowires, which provided improved detection capabilities for metronidazole [110], arsenic [111], NO_3_^−^ [29] and glucose [112]. NEAs of gold [113] or BDD [114] were successfully used for the analyses of dopamine and related compounds. Properties of TiO_2_ nanotube arrays have been exploited to detect trace level of the hair dye basic-brown 17 [115].

Both NEEs and NEAs have been functionalized to develop nanoelectrode-based biosensors suitable for advanced molecular diagnostics (see Table 2). In some cases, the functionalization of nanowire electrodes was performed. For instance, Au-coated silicon nanowires were functionalized with artificial peptides to detect RNA as response element for HIV-1 diagnostics [116]. Anti prostate-specific antigen (PSA) was electrodeposited with polypyrrole on 3D-gold nanowires to develop a label free immunosensor for PSA antigen [117]. 3D-gold NEEs modified with molecular imprinted poly(o-phenylenediamine) (PoPD) were exploited for aflatoxin B1 detection, reaching a detection limit of 19 fg·mL^−1^ by electrochemical impedance spectroscopy [118]. Molecular imprinted polyphenol was deposited on ensembles of Au-nanowires to develop a sensor for epithelial ovarian cancer antigen-125 [119]. 

The strategy based on the immobilization of the biorecognition layer on the polycarbonate of 2D-NEEs was applied to detect DNA hybridization [120]. In particular, the effect of the possible activation of the PC surface by oxidation with KMnO_4_ on the immobilization of SS-DNA was examined. It was shown that this treatment increases the number of reactive COOH groups present on the PC. However, it reflects in an increase in the capacitive current, due to partial desealing of the PC surrounding the gold nanodisks. Therefore care must be put in balancing the two effects to optimize the sensor’s performances [120]. A novel immunosensor for celiac disease diagnostic was based on electrogenerated chemiluminescence (ECL) at NEEs [121]. The target analyte, namely tissue transglutaminase antibody (anti-tTG) was captured by tissue transglutaminase (tTG) immobilized on the PC of the track-etched template membrane. Interaction of the target analyte with a suitable secondary antibody functionalized with a ruthenium label allowed quantification of anti-tTG. This was possible by ECL generation via the electrochemical oxidation of tripropylammine at the nanoelectrodes, this molecule acting both as co-reactant and redox mediator. A wide dynamic range and a low detection limit (0.5 ng·mL^−1^) characterized the sensor, which has been successfully applied in real samples such as blood serum of celiac patients. The PC surface of NEEs has been exploited also for immobilization of glucose oxidase in a miniaturized NEE-based enzymatic glucose sensor [54].

**Figure 14:**
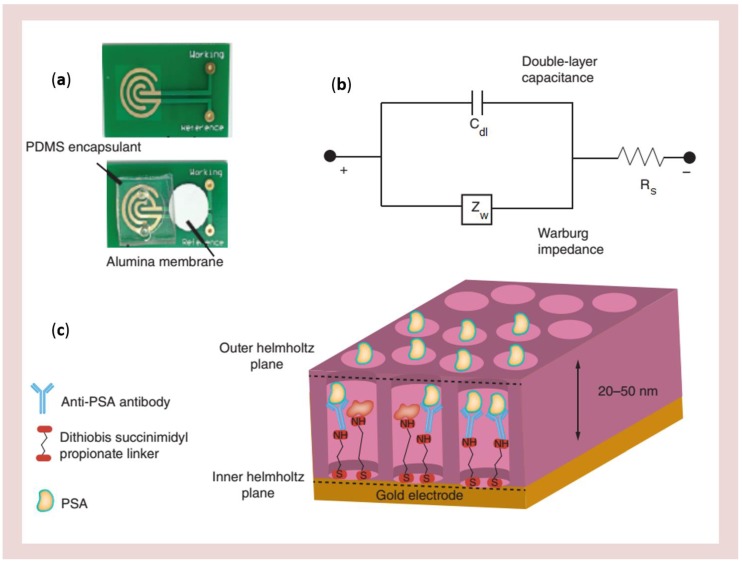
Electrical nanowell biosensor design, assembly and operation. Sensor device and operation (**a**) optical micrograph showing the gold microelectrodes, PDMS encapsulant and nanoporous alumina membrane (**b**) modified Randles equivalent circuit for label-free non-faradaic impedance spectroscopy and (**c**) schematic representation of binding events at the electrical double layer. PDMS: Polydimethyl siloxane; PSA: Prostate-specific antigen (reprinted with permission from [125]).

Among these recent advances, a particularly novel research line focuses on the study of array of nanochannels for performing the real-time monitoring of the enzyme reaction kinetics in confined nanospace (see Table 3). Concerning nanowells and nanochannels electrodes, different strategies for their fabrication were proposed. The most widely used material has been porous anodic alumina (PAA). PAA membranes are indeed characterized by tunable nanopore diameter in highly ordered array, easy surface modification, good mechanical stability and biocompatibility. A PAA nanowell based disposable biosensor for detecting PSA in human serum has been fabricated and tested [125]. It was designed by integrating nanoporous alumina membranes onto printed circuit board platforms, resulting in arrays of high-density nanowells, with gold electrodes at the bottom of the wells (Figure 14).

The sensor showed a rapid response time (<3 min) with a detection sensitivity in the pg·mL^−1^ range, for samples of small volume (~100 μL per test).A nanochannel–enzyme system was prepared by covalently linking glucose oxidase (GOD) on the inner wall of the nanochannels of a PAA membrane [123]. An Au disk was attached at the end of the PAA membrane and used as working electrode for the detection of H_2_O_2_ generated by the enzymatic reaction.

Continuing on this research line, Liu et al. proposed a nanochannel-based electrochemical reactor and a model to describe the kinetics of immunological reaction within nanochannels [124] Attention was put in investigating the role of steric and electrostatic effects on the flow parameters. 

A new approach to prepare nanopore arrays by alternate current (AC) electrodeposition of gold nanowires in PAA was proposed by Li et al. [127]. It was demonstrated that the voltammetric limiting current can be tuned by controlling the surface charge on the PAA walls by changing the solution pH.

A nanotube array enzymatic reactor was produced by electrostatically adsorbing cytochrome P450 2C9 enzyme (CYP2C9) on the inner wall of TiO_2_ nanotube arrays (TNAs) [126]. Different dimensions of TNAs were fabricated by controlling anodization potential or time. Au nanoparticles were deposited on the inner wall of TNAs to lower the electrical resistance. The CYP2C9 enzyme confined in the TNA exhibited excellent enzymatic activity, high affinity, and metabolic efficiency toward the substrate of tolbutamide with high sensitivity.

Future developments of such nanochannel–enzyme systems can be the design of future biosensors and enzyme reactors characterized by significantly high sensitivity and analytical efficiency.

## 8. Conclusions and Prospects

Nanoelectrode arrays and ensembles are opening new applicative prospects to the development of highly sensitive, selective and miniaturized biosensing devices. On one side, the template synthesis has made widely accessible the preparation of electrode systems with critical dimensions in the domain of nanometer. The use of nanoporous templates constitutes indeed an attractive and practical methodology for the preparation of a variety of ensembles of nanomaterials such as nanodisks, nanowires, nanotubes but also nanopores, nanowells or other hierarchically engineered nanostructures. These nanomaterials show particular and advantageous performances in the electroanalytical and biosensing fields. On the other hand, more sophisticated and complex procedures such as electron-beam or ion-beam lithography, allow the preparation of highly ordered arrays with perfectly controlled geometries. Both arrays and ensembles can be turned into 3D-nanostructures by using suitable nanofabrication procedures such as chemical or physical etching. 

For electroanalytical and sensing applications both NEEs/NEAs show dramatically enhanced signal-to-background ratios with respect to any other electrode system, together with the possibility to measure rather easily very fast charge transfer kinetic constant of interest for fundamental studies. However, a deep knowledge of the characteristics of diffusion and charge transfer processes at bio-nanostructured surfaces is required to obtain the maximum in biosensing performances from these devices. For instance, the use of too densely packed arrays of nanoelectrodes could hamper some of the advantages of NEEs/NEAs (e.g., highly improved S/N ratios). Moreover, wise users should take into account that the high sensitivity of these devices to the electron transfer kinetics make their analytical application advantageous mainly for very fast redox couples. 

For biosensing purposes, the smart use of the morphological characteristics and the composite structure of the arrays allow to maximize their biorecognition performances. This is the case when the large surface of 3D arrays is used to immobilize large amounts of biorecognition molecules. An interesting alternative, which exploits and keep at the maximum the high S/N ratio typical of NEEs/NEAs, is offered by the capability to functionalize the polymer surface which separates the nanoelectrodes in 2D arrays.

Practical applications for biosensing and diagnostic aims in real samples are growing quickly. To overcome problems of fouling, one can indeed protect the nanoelectrode surface by the clever use of thiols as anti-fouling agents [8]. New prospects will be opened by combining a deeper understanding of the mechanisms (and their modeling) which rule mass transport, (bio)chemical kinetics and electron transfer at NEEs/NEAs together with the development of more refined nanofabrication procedures.

From a technical development viewpoint, multiplexed analysis of different analytes will be possible by further developments in the preparation and application of individually addressable arrays of nanoelectrodes, as described in some frontier papers [94,128,129,130,131].

## Figures and Tables

**Table 1:** Articles published in the years 2012–2016, dealing with the application of NEEs/NEAs for the direct detection of small molecules of biological interest.

Nanosensor Type	Nanosense Platform	Target	Notes	Ref.
NEEs	Ensembles of copper nanowire electrodes	NO_3_^−^	Sensor durability and reproducibility are achieved by using a thin Nafion interlayer	[29]
NEEs	3D-gold nanotubes	Metronidazole	DL 0.1 nM	[110]
NEEs	3D-ensembles of gold nanowires electrodes	Inorganic Arsenic	Anodic stripping voltammetric determination of As(III), DL 0.08 µg·L^−1^, linear range up to 20 µg·L^−1^	[111]
NEAs	3D- ordered freestanding porous platinum (Pt) nanowire array electrode	Glucose, H_2_O_2_	Effect of granular and rougher porous nanowire surface on the bioactivity of glucose oxidase is examined	[112]
NEAs	Recessed NEAs with polymethylmethacrylate coated gold planar electrodes	Dopamine, Ascorbic acid, Uric acid	Sensing platform for detection of components in a mixture of analytes	[113]
NEAs	Nanocrystalline boron-doped diamond nanoelectrode arrays(BDD-NEAs)	Dopamine	Appropriate termination by choosing oxygen (O-) terminated BDD-NEAs, DL 100 nM	[114]
NEAs	Self-organized Ti/TiO_2_ nanotubular array	Hair dye basic brown 17	DL 1.3 × 10^−7^ M	[115]
NEAs	Pt nanoband electrode	Detection of, ferrocene carboxylic acid, hydrogen peroxide and 4-aminophenol	Chronoamperometric and cyclic voltammetric detection	[122]

**Table 2:** Articles published in the years 2012–2016, dealing with the application of NEEs/NEAs for the development of electrochemical sensors for biomacromolecules.

Nanosensor Type	Nanosense Platform	Target	Notes	Ref.
NEEs	Nonconductive PC component of the NEE is used for immobilizing glucose oxidase	Glucose	DL 36 µM	[54]
NEEs	Capturing proteins by interaction with the PC membrane of the NEE	Immunoglobulin IgY	Application to identify hen’s egg yolk in tempera paintings	[109]
NEAs	Au-coated vertical silicon nanowire electrode array	HIV-1 Rev response element (RRE) RNA	Immobilized artificial peptides for the recognition of HIV-1 RRE. DL 1.513 fM	[116]
NEAs	3D -gold nanowire array modified with electrodeposition of anti-PSA-doped Ppy polymers	Prostate-specific antigen	Linear response: 10 fg·mL^−1^ to 10 ng·mL^−1^, DL 0.3 fg·mL^−1^	[117]
NEEs	3D-gold nanoelectrode ensembles modified with poly- (o-phenylenediamine)	Aflatoxin B1	Cyclic voltammetry and electrochemical impedance spectroscopy have been employed. DL 0.019 ng·mL^−1^	[118]
NEEs	3D-gold nanoelectrode ensembles (3D-NEEs) modified with molecular imprinted polyphenol	Epithelial ovarian cancer antigen-125 (CA 125)	DL 0.5 U·mL^−1^	[119]
NEEs	Polymer surface of nanoelectrode ensembles bio-functionalized with DNA	DNA hybridization	Effect of the functionalization of the NEEs with the ss-DNA probe by measuring the changes in the methylene blue reduction signal is studied	[120]
EEs	Au nanodisk electrodes act as electrochemical transducers for initiating the ECL emission while PC is exploited for biorecogniton and to bind the luminescent label	Celiac disease diagnosis	Direct oxidation of tri-n-propylamine at NEEs generates ECL by a ruthenium label bound on antibody, DL 1.5 ng·mL^−1^. Application to human serum analysis	[121]

**Table 3:** Articles published in the years 2012–2016, dealing with biosensing application of arrays of nanochannels, nanopores and nanotubes.

Nanosensor Type	Nanosense Platform	Target	Notes	Ref.
Nano channel	Inner walls of the PAA nanochannels are functionalized with GOx; a bottom Au layer acts as working electrode.	Enzymatic reactivity of glucose oxidase	Activity and stability of the glucose oxidase immobilized in the nanochannels is largely enhanced	[123]
Nano channel	Functionalization of PAA nanochannels with DNA	To monitor online immunological reactions and biosensing process in the nanochannels	Study to evaluate the speed of antibody and the immunological reaction progress in nanochannels	[124]
Nanowell array	Integration of PAA membranes on printed circuit board platforms	Prostate-specific antigen (PSA)	PSA detection between 0.01 and 1000 ng·mL^−1^	[125]
Nanotube array	Electrodepositing Au nanoparticles on the inner wall of TiO_2_ nanotube arrays	Cytochrome P450 2C9 enzyme as a model enzyme and tolbutamide as a model substrate	Excellent enzymatic activity, high affinity, and metabolic efficiency for tolbutamide	[126]
Nanopore array	Electrodeposition of gold nanowires in porous anodic alumina membranes by alternating current	Investigation of the electrochemistry property of nanopore array electrode	Voltammetric limiting current can be regulated by surface charge changes on the PAA walls by changing the solution pH	[127]
